# [2-(3,5-Dimethyl-1*H*-pyrazol-1-yl-κ*N* ^2^)-1,10-phenanthroline-κ^2^ *N*,*N*′]bis­(thio­cyanato-κ*N*)cadmium(II)

**DOI:** 10.1107/S1600536809039920

**Published:** 2009-10-07

**Authors:** Yu Qing Wang, Lin Meng, Jing Min Shi

**Affiliations:** a Department of Chemistry, Shandong Normal University, Jinan 250014, People’s Republic of China

## Abstract

In the title complex, [Cd(NCS)_2_(C_17_H_14_N_4_)], the Cd^II^ ion is in a distorted trigonal-bipyramidal CdN_5_ coordination geometry. In the crystal structure, there is a π–π stacking inter­action involving a pyrazole ring and a symmetry-related pyridine ring with a centroid–centroid distance of 3.578 (3) Å.

## Related literature

For a related structure, see: Liu *et al.* (2008 ▶).
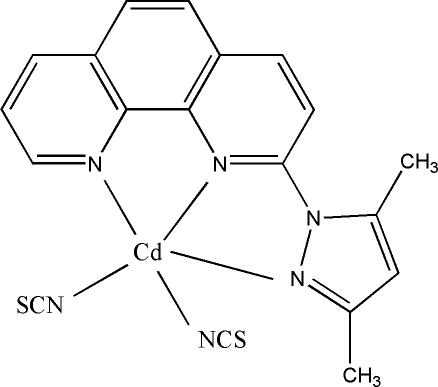


## Experimental

### 

#### Crystal data

[Cd(NCS)_2_(C_17_H_14_N_4_)]
*M* *_r_* = 502.88
Monoclinic, 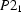

*a* = 7.7324 (15) Å
*b* = 14.811 (3) Å
*c* = 8.7150 (17) Å
β = 104.006 (2)°
*V* = 968.4 (3) Å^3^
*Z* = 2
Mo *K*α radiation
μ = 1.36 mm^−1^
*T* = 298 K
0.25 × 0.16 × 0.10 mm

#### Data collection

Bruker SMART APEX CCD diffractometer
Absorption correction: multi-scan (*SADABS*; Sheldrick, 1996 ▶) *T*_min_ = 0.727, *T*_max_ = 0.876
5622 measured reflections
3971 independent reflections
3695 reflections with *I* > 2σ(*I*)
*R*_int_ = 0.021

#### Refinement

*R*[*F* ^2^ > 2σ(*F* ^2^)] = 0.037
*wR*(*F* ^2^) = 0.082
*S* = 1.03
3971 reflections
255 parameters
1 restraint
H-atom parameters constrained
Δρ_max_ = 0.90 e Å^−3^
Δρ_min_ = −0.38 e Å^−3^
Absolute structure: Flack (1983 ▶), 1803 Friedel pairs
Flack parameter: 0.03 (3)

### 

Data collection: *SMART* (Bruker, 1997 ▶); cell refinement: *SAINT* (Bruker, 1997 ▶); data reduction: *SAINT*; program(s) used to solve structure: *SHELXTL* (Sheldrick, 2008 ▶); program(s) used to refine structure: *SHELXTL*; molecular graphics: *SHELXTL*; software used to prepare material for publication: *SHELXTL*.

## Supplementary Material

Crystal structure: contains datablocks I, global. DOI: 10.1107/S1600536809039920/lh2919sup1.cif

Structure factors: contains datablocks I. DOI: 10.1107/S1600536809039920/lh2919Isup2.hkl

Additional supplementary materials:  crystallographic information; 3D view; checkCIF report

## Figures and Tables

**Table:** 

Cd1—N5	2.148 (4)
Cd1—N4	2.174 (5)
Cd1—N2	2.286 (4)
Cd1—N3	2.310 (4)
Cd1—N1	2.350 (4)

**Table:** 

N5—Cd1—N4	104.40 (19)
N5—Cd1—N2	130.96 (16)
N4—Cd1—N2	124.53 (18)
N5—Cd1—N3	103.47 (16)
N4—Cd1—N3	99.06 (19)
N2—Cd1—N3	68.42 (14)
N5—Cd1—N1	103.92 (15)
N4—Cd1—N1	101.66 (18)
N2—Cd1—N1	71.66 (14)
N3—Cd1—N1	140.03 (13)

**Fig. 1.**
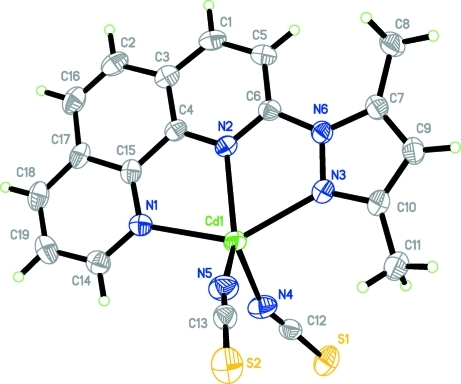
The molecular structure of title compound with the atom-numbering scheme. Displacement ellipsoids are drawn at the 30% probability level.

## supplementary crystallographic information

### Comment

Derivatives of 1,10-phenanthroline play an important role in modern coordination chemistry and many complexes have been reported with these types of compounds as ligands [see e.g. Liu et al. (2008) for a closely related Cd complex]. To the best of knowledge, no crystal structures of complexes with 2-(3,5-Dimethyl-1H-pyrazol-1-yl)-1,10-phenanthroline as a ligand have been reported so far, and herein we report the crystal structure of the title compound (I).

The molecular structure of the title compound in shown in Fig. 1. The Cd^II^ ion is coordinated by five N atoms in a distorted trigonal bipyramidal environment. Generally, Cd^II^ ion assumes six atoms coordination mode and the present five coordination mode may be attributed to the chelation mode of the ligand 2-(3,5-Dimethyl-1H-pyrazol-1-yl)-1,10-phenanthroline. The non-hydrogen atoms of the 2-(3,5-Dimethyl-1H-pyrazol-1-yl)-1,10-phenanthroline ligand define a plane within 0.0705 Å with a maximum deviation of 0.189 (6) Å for atom C8. In the crystal structure, there is a π–π stacking interaction involving the pyrazole ring and a symmetry related pyridine ring with the relevant distances being  Cg1···Cg2^i^ = 3.578 (3) Å and Cg1···Cg2^i^_perp_ = 3.361 Å (symmetry code: (I) -1+x, y, z; Cg1 and Cg2 are the centroids of C7-C10/N3/N6 pyrazol ring and N1/C14/15/C17-C19 pyridine ring, respectively; Cg1···Cg2^i^_perp_ is the perpendicular distance from Cg1 ring to Cg2^i^ ring).

### Experimental

10 ml methanol solution of 2-(3,5-Dimethyl-1*H*-pyrazol-1-yl)-1,10-phenanthroline (0.0406 g, 0.148 mmol) was added to a 10 ml methanol solution containing Cd(ClO_4_).6H_2_O (0.0655 g, 0.156 mmol) and NaNCS (0.0121 g, 0.149 mmol), and the mixed soluton was stirred for a few minutes. The colorless single crystals were obtained after the filtrate had been allowed to stand at room temperature for about a week.

### Refinement

All H atoms were placed in calculated positions and refined as riding with C—H = 0.96 Å, *U*_iso_ = 1.5*U*_eq_(C) for methyl H and C—H = 0.93 Å, *U*_iso_ = 1.2*U*_eq_(C) for other H atoms.

### Crystal data

**Table:**

[Cd(NCS)_2_(C_17_H_14_N_4_)]	*F*(000) = 500
*M**_r_* = 502.88	*D*_x_ = 1.725 Mg m^−^^3^
Monoclinic, *P*2_1_	Mo *K*α radiation, λ = 0.71073 Å
Hall symbol: P 2yb	Cell parameters from 2683 reflections
*a* = 7.7324 (15) Å	θ = 2.4–24.9°
*b* = 14.811 (3) Å	µ = 1.36 mm^−^^1^
*c* = 8.7150 (17) Å	*T* = 298 K
β = 104.006 (2)°	Block, colorless
*V* = 968.4 (3) Å^3^	0.25 × 0.16 × 0.10 mm
*Z* = 2	

### Data collection

**Table:**

Bruker SMART APEX CCD diffractometer	3971 independent reflections
Radiation source: fine-focus sealed tube	3695 reflections with *I* > 2σ(*I*)
graphite	*R*_int_ = 0.021
φ and ω scans	θ_max_ = 27.0°, θ_min_ = 2.4°
Absorption correction: multi-scan (*SADABS*; Sheldrick, 1996)	*h* = −9→9
*T*_min_ = 0.727, *T*_max_ = 0.876	*k* = −18→18
5622 measured reflections	*l* = −11→4

### Refinement

**Table:**

Refinement on *F*^2^	Secondary atom site location: difference Fourier map
Least-squares matrix: full	Hydrogen site location: inferred from neighbouring sites
*R*[*F*^2^ > 2σ(*F*^2^)] = 0.037	H-atom parameters constrained
*wR*(*F*^2^) = 0.082	*w* = 1/[σ^2^(*F*_o_^2^) + (0.0366*P*)^2^ + 0.2846*P*] where *P* = (*F*_o_^2^ + 2*F*_c_^2^)/3
*S* = 1.03	(Δ/σ)_max_ = 0.043
3971 reflections	Δρ_max_ = 0.90 e Å^−^^3^
255 parameters	Δρ_min_ = −0.38 e Å^−^^3^
1 restraint	Absolute structure: Flack (1983), 1803 Friedel pairs
Primary atom site location: structure-invariant direct methods	Flack parameter: 0.03 (3)

### Special details

**Table:**

Geometry. All e.s.d.'s (except the e.s.d. in the dihedral angle between two l.s. planes) are estimated using the full covariance matrix. The cell e.s.d.'s are taken into account individually in the estimation of e.s.d.'s in distances, angles and torsion angles; correlations between e.s.d.'s in cell parameters are only used when they are defined by crystal symmetry. An approximate (isotropic) treatment of cell e.s.d.'s is used for estimating e.s.d.'s involving l.s. planes.
Refinement. Refinement of *F*^2^ against ALL reflections. The weighted *R*-factor *wR* and goodness of fit *S* are based on *F*^2^, conventional *R*-factors *R* are based on *F*, with *F* set to zero for negative *F*^2^. The threshold expression of *F*^2^ > σ(*F*^2^) is used only for calculating *R*-factors(gt) *etc*. and is not relevant to the choice of reflections for refinement. *R*-factors based on *F*^2^ are statistically about twice as large as those based on *F*, and *R*- factors based on ALL data will be even larger.

### Fractional atomic coordinates and isotropic or equivalent isotropic displacement parameters (Å^2^)

**Table:**

	*x*	*y*	*z*	*U*_iso_*/*U*_eq_	
C1	0.6366 (8)	0.9188 (4)	0.3905 (6)	0.0571 (13)	
H1	0.6346	0.9687	0.4547	0.069*	
C2	0.9604 (10)	0.8931 (5)	0.5143 (8)	0.065 (2)	
H2	0.9658	0.9428	0.5804	0.078*	
C3	0.7957 (8)	0.8709 (4)	0.4056 (6)	0.0537 (13)	
C4	0.7877 (6)	0.7957 (3)	0.3077 (5)	0.0422 (10)	
C5	0.4839 (8)	0.8938 (3)	0.2833 (7)	0.0555 (13)	
H5	0.3786	0.9259	0.2749	0.067*	
C6	0.4893 (8)	0.8186 (3)	0.1861 (6)	0.0426 (12)	
C7	0.1719 (6)	0.8183 (3)	0.0153 (6)	0.0505 (12)	
C8	0.0992 (8)	0.9038 (4)	0.0664 (8)	0.0646 (15)	
H8A	−0.0189	0.9140	0.0024	0.097*	
H8B	0.1747	0.9534	0.0543	0.097*	
H8C	0.0953	0.8988	0.1754	0.097*	
C9	0.0877 (7)	0.7584 (3)	−0.0940 (7)	0.0547 (13)	
H9	−0.0289	0.7623	−0.1547	0.066*	
C10	0.2094 (7)	0.6895 (4)	−0.0984 (6)	0.0498 (12)	
C11	0.1863 (8)	0.6062 (4)	−0.1965 (8)	0.0699 (17)	
H11A	0.2961	0.5921	−0.2240	0.105*	
H11B	0.0943	0.6158	−0.2911	0.105*	
H11C	0.1533	0.5570	−0.1377	0.105*	
C12	0.5035 (9)	0.4510 (4)	0.1563 (7)	0.0515 (14)	
C13	0.7029 (6)	0.6142 (3)	−0.3031 (6)	0.0472 (12)	
C14	1.0787 (7)	0.6191 (3)	0.2318 (6)	0.0537 (14)	
H14	1.0711	0.5689	0.1664	0.064*	
C15	0.9446 (6)	0.7413 (3)	0.3156 (5)	0.0440 (10)	
C16	1.1065 (8)	0.8435 (5)	0.5223 (7)	0.0664 (16)	
H16	1.2123	0.8603	0.5928	0.080*	
C17	1.1048 (7)	0.7655 (3)	0.4256 (6)	0.0519 (12)	
C18	1.2533 (7)	0.7108 (4)	0.4316 (6)	0.0590 (14)	
H18	1.3609	0.7240	0.5026	0.071*	
C19	1.2406 (7)	0.6379 (6)	0.3332 (6)	0.0634 (14)	
H19	1.3395	0.6018	0.3348	0.076*	
Cd1	0.64744 (4)	0.64456 (2)	0.05473 (3)	0.04668 (10)	
N1	0.9318 (6)	0.6684 (2)	0.2214 (4)	0.0444 (10)	
N2	0.6373 (6)	0.7719 (3)	0.2006 (4)	0.0405 (9)	
N3	0.3643 (5)	0.7057 (3)	0.0005 (5)	0.0476 (9)	
N4	0.5860 (8)	0.5095 (3)	0.1234 (7)	0.0668 (14)	
N5	0.6979 (6)	0.6273 (3)	−0.1753 (5)	0.0603 (12)	
N6	0.3425 (5)	0.7858 (2)	0.0737 (5)	0.0441 (9)	
S1	0.3932 (3)	0.36735 (13)	0.2031 (2)	0.0779 (5)	
S2	0.7124 (2)	0.59068 (13)	−0.48295 (17)	0.0695 (4)	

### Atomic displacement parameters (Å^2^)

**Table:**

	*U*^11^	*U*^22^	*U*^33^	*U*^12^	*U*^13^	*U*^23^
C1	0.076 (4)	0.043 (3)	0.054 (3)	−0.003 (3)	0.020 (3)	−0.011 (2)
C2	0.079 (6)	0.060 (3)	0.053 (3)	−0.016 (4)	0.009 (3)	−0.017 (3)
C3	0.062 (3)	0.049 (3)	0.052 (3)	−0.007 (2)	0.016 (2)	−0.003 (2)
C4	0.049 (3)	0.039 (2)	0.041 (2)	−0.0077 (19)	0.016 (2)	0.0044 (18)
C5	0.064 (4)	0.043 (3)	0.061 (3)	0.007 (2)	0.019 (3)	−0.003 (2)
C6	0.048 (3)	0.036 (2)	0.046 (3)	−0.002 (2)	0.016 (2)	0.006 (2)
C7	0.043 (3)	0.046 (3)	0.064 (3)	−0.001 (2)	0.016 (2)	0.015 (2)
C8	0.053 (3)	0.049 (3)	0.093 (5)	0.007 (2)	0.019 (3)	0.015 (3)
C9	0.041 (3)	0.056 (3)	0.065 (3)	0.000 (2)	0.008 (2)	0.011 (2)
C10	0.045 (3)	0.054 (3)	0.050 (3)	−0.009 (2)	0.010 (2)	−0.002 (2)
C11	0.055 (3)	0.078 (4)	0.074 (4)	−0.010 (3)	0.010 (3)	−0.018 (3)
C12	0.053 (3)	0.052 (3)	0.047 (3)	0.010 (3)	0.007 (2)	−0.008 (2)
C13	0.041 (3)	0.042 (2)	0.057 (3)	−0.0006 (18)	0.010 (2)	0.0041 (19)
C14	0.058 (3)	0.053 (3)	0.055 (3)	0.002 (2)	0.022 (2)	0.005 (2)
C15	0.044 (3)	0.050 (3)	0.040 (2)	−0.005 (2)	0.0133 (19)	0.011 (2)
C16	0.059 (4)	0.073 (4)	0.059 (3)	−0.016 (3)	−0.003 (3)	−0.001 (3)
C17	0.047 (3)	0.056 (3)	0.050 (3)	−0.016 (2)	0.006 (2)	0.008 (2)
C18	0.045 (3)	0.076 (4)	0.050 (3)	−0.010 (3)	−0.001 (2)	0.012 (3)
C19	0.049 (3)	0.075 (4)	0.069 (3)	0.013 (4)	0.019 (2)	0.020 (4)
Cd1	0.04711 (18)	0.04536 (16)	0.04917 (17)	−0.0017 (2)	0.01472 (12)	−0.0109 (2)
N1	0.051 (2)	0.042 (3)	0.0419 (19)	−0.0003 (15)	0.0143 (16)	0.0038 (15)
N2	0.046 (2)	0.0389 (19)	0.036 (2)	−0.0045 (17)	0.0089 (17)	−0.0002 (16)
N3	0.046 (2)	0.049 (2)	0.046 (2)	−0.0052 (17)	0.0088 (17)	−0.0023 (18)
N4	0.085 (4)	0.044 (3)	0.074 (3)	−0.007 (3)	0.025 (3)	−0.001 (2)
N5	0.072 (3)	0.059 (4)	0.055 (2)	−0.005 (2)	0.026 (2)	−0.011 (2)
N6	0.041 (2)	0.043 (2)	0.050 (2)	−0.0024 (16)	0.0136 (17)	0.0030 (17)
S1	0.0718 (11)	0.0883 (12)	0.0750 (11)	−0.0273 (9)	0.0203 (8)	−0.0011 (9)
S2	0.0666 (9)	0.1010 (12)	0.0424 (7)	0.0002 (8)	0.0160 (6)	0.0019 (7)

### Geometric parameters (Å, °)

**Table:**

C1—C5	1.367 (8)	C11—H11B	0.9600
C1—C3	1.398 (9)	C11—H11C	0.9600
C1—H1	0.9300	C12—N4	1.152 (8)
C2—C16	1.335 (11)	C12—S1	1.611 (7)
C2—C3	1.429 (9)	C13—N5	1.141 (6)
C2—H2	0.9300	C13—S2	1.624 (6)
C3—C4	1.396 (7)	C14—N1	1.335 (6)
C4—N2	1.350 (6)	C14—C19	1.375 (8)
C4—C15	1.444 (7)	C14—H14	0.9300
C5—C6	1.407 (7)	C15—N1	1.346 (6)
C5—H5	0.9300	C15—C17	1.416 (7)
C6—N2	1.316 (7)	C16—C17	1.429 (8)
C6—N6	1.395 (7)	C16—H16	0.9300
C7—C9	1.348 (7)	C17—C18	1.395 (8)
C7—N6	1.380 (6)	C18—C19	1.368 (9)
C7—C8	1.496 (8)	C18—H18	0.9300
C8—H8A	0.9600	C19—H19	0.9300
C8—H8B	0.9600	Cd1—N5	2.148 (4)
C8—H8C	0.9600	Cd1—N4	2.174 (5)
C9—C10	1.396 (8)	Cd1—N2	2.286 (4)
C9—H9	0.9300	Cd1—N3	2.310 (4)
C10—N3	1.317 (6)	Cd1—N1	2.350 (4)
C10—C11	1.486 (7)	N3—N6	1.377 (5)
C11—H11A	0.9600		
			
C5—C1—C3	121.5 (5)	N1—C14—H14	118.0
C5—C1—H1	119.3	C19—C14—H14	118.0
C3—C1—H1	119.3	N1—C15—C17	122.6 (5)
C16—C2—C3	121.0 (6)	N1—C15—C4	118.8 (4)
C16—C2—H2	119.5	C17—C15—C4	118.6 (4)
C3—C2—H2	119.5	C2—C16—C17	121.8 (6)
C4—C3—C1	116.0 (5)	C2—C16—H16	119.1
C4—C3—C2	119.3 (6)	C17—C16—H16	119.1
C1—C3—C2	124.7 (6)	C18—C17—C15	117.1 (5)
N2—C4—C3	122.3 (5)	C18—C17—C16	123.9 (5)
N2—C4—C15	117.5 (4)	C15—C17—C16	119.0 (5)
C3—C4—C15	120.2 (4)	C19—C18—C17	120.1 (5)
C1—C5—C6	118.6 (5)	C19—C18—H18	120.0
C1—C5—H5	120.7	C17—C18—H18	120.0
C6—C5—H5	120.7	C18—C19—C14	118.6 (6)
N2—C6—N6	115.2 (5)	C18—C19—H19	120.7
N2—C6—C5	120.7 (5)	C14—C19—H19	120.7
N6—C6—C5	124.1 (5)	N5—Cd1—N4	104.40 (19)
C9—C7—N6	106.7 (5)	N5—Cd1—N2	130.96 (16)
C9—C7—C8	128.0 (5)	N4—Cd1—N2	124.53 (18)
N6—C7—C8	125.4 (5)	N5—Cd1—N3	103.47 (16)
C7—C8—H8A	109.5	N4—Cd1—N3	99.06 (19)
C7—C8—H8B	109.5	N2—Cd1—N3	68.42 (14)
H8A—C8—H8B	109.5	N5—Cd1—N1	103.92 (15)
C7—C8—H8C	109.5	N4—Cd1—N1	101.66 (18)
H8A—C8—H8C	109.5	N2—Cd1—N1	71.66 (14)
H8B—C8—H8C	109.5	N3—Cd1—N1	140.03 (13)
C7—C9—C10	106.7 (5)	C14—N1—C15	117.5 (4)
C7—C9—H9	126.7	C14—N1—Cd1	127.8 (3)
C10—C9—H9	126.7	C15—N1—Cd1	114.7 (3)
N3—C10—C9	111.1 (5)	C6—N2—C4	120.8 (4)
N3—C10—C11	119.5 (5)	C6—N2—Cd1	121.7 (3)
C9—C10—C11	129.4 (5)	C4—N2—Cd1	117.4 (3)
C10—C11—H11A	109.5	C10—N3—N6	105.5 (4)
C10—C11—H11B	109.5	C10—N3—Cd1	136.8 (4)
H11A—C11—H11B	109.5	N6—N3—Cd1	117.3 (3)
C10—C11—H11C	109.5	C12—N4—Cd1	158.7 (5)
H11A—C11—H11C	109.5	C13—N5—Cd1	171.3 (4)
H11B—C11—H11C	109.5	N3—N6—C7	110.0 (4)
N4—C12—S1	178.4 (6)	N3—N6—C6	117.1 (4)
N5—C13—S2	177.3 (5)	C7—N6—C6	132.9 (4)
N1—C14—C19	124.1 (5)		
			
C5—C1—C3—C4	−1.3 (8)	C5—C6—N2—Cd1	176.5 (4)
C5—C1—C3—C2	179.8 (6)	C3—C4—N2—C6	−1.2 (7)
C16—C2—C3—C4	0.7 (9)	C15—C4—N2—C6	−179.6 (4)
C16—C2—C3—C1	179.5 (6)	C3—C4—N2—Cd1	−178.6 (4)
C1—C3—C4—N2	2.2 (7)	C15—C4—N2—Cd1	3.0 (5)
C2—C3—C4—N2	−178.8 (5)	N5—Cd1—N2—C6	88.0 (4)
C1—C3—C4—C15	−179.4 (5)	N4—Cd1—N2—C6	−87.5 (4)
C2—C3—C4—C15	−0.5 (7)	N3—Cd1—N2—C6	−1.3 (4)
C3—C1—C5—C6	−0.5 (9)	N1—Cd1—N2—C6	−179.2 (4)
C1—C5—C6—N2	1.6 (8)	N5—Cd1—N2—C4	−94.6 (4)
C1—C5—C6—N6	179.3 (5)	N4—Cd1—N2—C4	89.9 (4)
N6—C7—C9—C10	0.8 (6)	N3—Cd1—N2—C4	176.1 (4)
C8—C7—C9—C10	−179.3 (5)	N1—Cd1—N2—C4	−1.9 (3)
C7—C9—C10—N3	−1.3 (6)	C9—C10—N3—N6	1.3 (6)
C7—C9—C10—C11	179.4 (5)	C11—C10—N3—N6	−179.3 (4)
N2—C4—C15—N1	−2.6 (6)	C9—C10—N3—Cd1	−170.8 (4)
C3—C4—C15—N1	179.0 (4)	C11—C10—N3—Cd1	8.6 (8)
N2—C4—C15—C17	179.2 (4)	N5—Cd1—N3—C10	46.2 (5)
C3—C4—C15—C17	0.8 (6)	N4—Cd1—N3—C10	−61.1 (5)
C3—C2—C16—C17	−1.2 (10)	N2—Cd1—N3—C10	175.3 (6)
N1—C15—C17—C18	1.0 (7)	N1—Cd1—N3—C10	178.4 (4)
C4—C15—C17—C18	179.2 (4)	N5—Cd1—N3—N6	−125.2 (3)
N1—C15—C17—C16	−179.3 (5)	N4—Cd1—N3—N6	127.5 (3)
C4—C15—C17—C16	−1.2 (7)	N2—Cd1—N3—N6	3.8 (3)
C2—C16—C17—C18	−178.9 (6)	N1—Cd1—N3—N6	6.9 (4)
C2—C16—C17—C15	1.5 (9)	S1—C12—N4—Cd1	−177 (100)
C15—C17—C18—C19	0.4 (8)	N5—Cd1—N4—C12	−114.8 (14)
C16—C17—C18—C19	−179.1 (5)	N2—Cd1—N4—C12	61.7 (15)
C17—C18—C19—C14	−1.3 (9)	N3—Cd1—N4—C12	−8.2 (14)
N1—C14—C19—C18	0.9 (9)	N1—Cd1—N4—C12	137.4 (14)
C19—C14—N1—C15	0.6 (7)	S2—C13—N5—Cd1	−84 (11)
C19—C14—N1—Cd1	180.0 (4)	N4—Cd1—N5—C13	61 (3)
C17—C15—N1—C14	−1.5 (6)	N2—Cd1—N5—C13	−115 (3)
C4—C15—N1—C14	−179.7 (4)	N3—Cd1—N5—C13	−42 (3)
C17—C15—N1—Cd1	179.0 (3)	N1—Cd1—N5—C13	167 (3)
C4—C15—N1—Cd1	0.9 (5)	C10—N3—N6—C7	−0.8 (5)
N5—Cd1—N1—C14	−49.9 (4)	Cd1—N3—N6—C7	173.1 (3)
N4—Cd1—N1—C14	58.3 (4)	C10—N3—N6—C6	179.9 (4)
N2—Cd1—N1—C14	−178.9 (4)	Cd1—N3—N6—C6	−6.1 (5)
N3—Cd1—N1—C14	178.0 (3)	C9—C7—N6—N3	0.0 (5)
N5—Cd1—N1—C15	129.5 (3)	C8—C7—N6—N3	−180.0 (5)
N4—Cd1—N1—C15	−122.3 (3)	C9—C7—N6—C6	179.1 (5)
N2—Cd1—N1—C15	0.5 (3)	C8—C7—N6—C6	−0.9 (9)
N3—Cd1—N1—C15	−2.5 (4)	N2—C6—N6—N3	4.9 (6)
N6—C6—N2—C4	−178.6 (4)	C5—C6—N6—N3	−172.8 (5)
C5—C6—N2—C4	−0.8 (7)	N2—C6—N6—C7	−174.1 (5)
N6—C6—N2—Cd1	−1.4 (6)	C5—C6—N6—C7	8.1 (9)
